# Novel energy balance tracking to support personalised AI health coaching: a real-world evaluation of the ENHANCE framework

**DOI:** 10.1038/s41366-026-02100-8

**Published:** 2026-05-12

**Authors:** Arthur B. Daw, Maxime Petit, Scott A. Willis, Lewis J. James, James A. King

**Affiliations:** 1 National Centre for Sport and Exercise Medicine, School of Sport, Exercise and Health Sciences, Loughborough University, Loughborough, UK; 2 Surpic Limited, Cheltenham, UK; 3 NIHR Leicester Biomedical Research Centre, University Hospitals of Leicester NHS Trust and University of Leicester, Leicester, UK

**Keywords:** Weight management, Lifestyle modification, Preventive medicine

## Abstract

**Background:**

Energy balance (EB) is the key determinant of fat gain, yet accurate EB tracking is difficult outside laboratory settings. Traditional methods are burdensome (e.g. food-logs) or lack daily resolution (e.g. body weight monitoring), limiting suitability for integration with free-living AI-powered health coaching.

**Objective:**

To introduce ENHANCE—a novel framework prioritising interpretability and temporal accuracy—and demonstrate its use as a low-burden, accurate method for tracking EB using smart devices and minimal self-report, suitable for AI coaching.

**Methods:**

This 4-week observational study spanned the Christmas to New Year 2024/25 festive period. Participants submitted daily blinded body weight measurements via Wi-Fi scales and EB-related questions via a mobile app, taking <2 min. Data were used to generate five weight trends: raw (from scales), smoothed (±3-day average), piecewise (3-segments), predicted (from EB), and corrected. The correction aligned predicted and smoothed trends, using proximity and noise-weighted adjustments, producing enhanced data for AI coaching. An end-of-study questionnaire assessed acceptability and behavioural reactivity.

**Results:**

Of 23 participants, 18 were analysed. Five were excluded due to illness (*n* = 4) or bereavement (*n* = 1). Participants completed 94% (5.1%) of body weight measurements and 100% of EB-related submissions. Questionnaire results showed low burden (1.8/5) and behavioural reactivity (1.5/5). Group-level predicted trends explained 90.4% of smoothed trend variance (R² = 0.904; mean absolute error [MAE]: 93 g). Corrected trends aligned more closely with piecewise segments than raw trends (MAE: 46 g vs 77 g). Individual-level mean EB corrections were +41 kcal/day—just 2% of reported intake. The corrected trend enhanced interpretability and plausibility while preserving real-world validity. Calculated mean net fat weight change during the monitoring phase was +0.8 kg (0.4 kg); mean net EB was +223 kcal/day (130 kcal/day).

**Conclusions:**

This scalable method delivers the accuracy and practicality needed for real-world EB tracking—laying the foundation for continuous personalised AI coaching.

## Introduction

Artificial Intelligence (AI) is transforming healthcare [1]. A promising application is personalised AI coaching apps that monitor health using smart device data, including physical activity, sleep, blood pressure, stress, and weight. Energy balance (EB) is often overlooked but is central to cardiometabolic health [2] and foundational to weight control models, embodying principles of energy conservation and regulatory biology [3]. Accurate free-living EB tracking is therefore an essential requirement of AI-based coaching platforms [4] yet remains challenging to measure [2].

EB can be estimated from body weight changes using predictive equations, providing a low-burden solution [5], but lacks the resolution needed for daily feedback. Conversely, daily EB tracking using estimates of energy intake (EI) (via food-tracking) and energy expenditure (EE) (via smart device data) improves precision but requires ongoing user engagement [6]. Although these methods are initially accepted, user engagement drops rapidly, limiting long-term effectiveness [7]. Sustained AI-based support requires reliable EB tracking, even during low-engagement high-risk periods [4], such as holidays when 0.5–1 kg fat gain is common [8]. To maximise the potential of AI coaching, valid methods are needed to reliably track EB year-round, including during high-risk periods [9, 10].

This report introduces:

1. **ENHANCE framework**: ENHANCE (Estimation and Normalisation for Holistic Alignment, Nuance, and Contextual Enhancements) improves tracking accuracy and interpretability by combining holistic temporal data with physiological and behavioural relationships. Its aim is to enhance user decision-making, engagement, and outcomes in free-living settings—not to generate a ground truth comparable to laboratory measurements.
2. **ENHANCE EB tracking**: A low-burden daily EB tracking method combining smart device data with minimal self-report, implemented using the ENHANCE framework.

We present the conceptual framework, implementation, and preliminary evaluation—delivered through the Twists.com AI coaching platform [11]—and evaluate its feasibility for continuous AI-based coaching in real-world settings.

## Methods

### Ethical approval

Ethical approval was granted by Loughborough University Ethics Advisory Committee (Ref: 21271) and the study complied with the Declaration of Helsinki [12]. All participants gave informed consent (digitally).

### Data architecture and anonymisation

Participants received a unique identification number and pseudonym during onboarding. Body weight and app data were transmitted over Wi-Fi and stored on a Microsoft database hosted on Amazon Web Services. Access was restricted to authorised researchers via a secure software-as-a-service interface. Questionnaire responses were collected via Google Forms and linked to participants via date of birth only.

### Study design and setting

This remote observational study was conducted between 08 December 2024 to 15 January 2025. The Christmas to New Year festive period is a high-risk EB disruptor—due to increased EI, reduced EE, and competing personal demands [8]—making it ideal to evaluate method performance.

### Study phases

1. **Onboarding (08 December–10 December)**: Participants connected their Wi-Fi scales, installed the Twists app, and estimated typical EB under habitual conditions.
2. **Baseline (08 December–14 December)**: Participants established a baseline body weight trend while familiarising themselves with the scales and app. Baseline data configured the platform but were excluded from results.
3. **Monitoring (15 December–10 January)**: Participants recorded blind daily body weight measurements and answered EB-related questions. Feedback was withheld to preserve naturalistic behaviour.
4. **Feedback (10 January–15 January)**: Participant acceptability and behavioural reactivity were assessed using an end-of-study multiple-choice questionnaire.

### Participants and recruitment

Participants were recruited online from Gloucestershire, UK. Inclusion criteria were: men and women, 20–49 years, BMI 18.5–29.9 kg/m², stable body weight (self-reported body mass change ±3 kg in the last 3-months), and not travelling overnight >1 night. Twelve is a recommended sample for feasibility [13], however, we enrolled 23 participants to allow for attrition and to support preliminary exploration of individual-level prediction variability. To preserve naturalistic behaviour, participants were only informed that the study was investigating how the festive period affects population EB. Weight monitoring was not discussed, and readings were blinded. An end-of-study questionnaire assessed behavioural reactivity.

### Data collection protocol

Participants received Wi-Fi-enabled scales and proprietary Twists app for daily EB-related submissions—both scales and app were developed by Surpic Limited. Twists provides structured, goal-oriented tracking and AI coaching, but was modified for this study to ensure participants remained blinded to EB and weight feedback. During onboarding, participants watched a demonstration video and received a text-based guide. Each morning, participants recorded blinded weight and self-reported the prior day’s EB-related data. Daily tasks took <2 min. Notifications were sent at 6:00 AM (silent)—for measurement reminders, 2:00 PM—if self-reports were missed, and 4:00 PM—if measurements were missed.

### Weight measurements via Wi-Fi scales

Each morning, participants weighed themselves after toileting, ideally nude or wearing consistent minimal clothing. Scales were placed on hard, flat surfaces. Displays confirmed measurement and Wi-Fi upload but did not show weight. The scales were manufactured by Surpic Limited (Cheltenham, UK) and assembled and UKCA-certified by Zhongshan Frecom Electronic Limited (Guangdong, China). Raw body weight data were recorded to 0.1 kg resolution. Scale accuracy was independently validated by Société Générale de Surveillance (Geneva, Switzerland), confirming ANSI/ASQ Z1.4 (2003, R2018) standards at AQL Level II [14], and precision ±0.2–0.3 kg [15].

### Self-reporting via mobile app

Three daily EB-related metrics were collected via the Twists app: eating (%), steps (count), and exercise (kcal). To minimise time burden and enhance resilience to device issues, participants could choose from multiple simple input methods per metric.

Eating was estimated as a percentage deviation from ‘typical’ intake. Participants were weight stable at onboarding, therefore typical EE was used to estimate typical EI. Participants either selected a deviation percentage from five preset options or entered a custom value from −100% to +100%. This method was intentionally approximate and not intended to provide precise estimates of EI, but to generate a plausible trend line with minimal input for further analysis.

The five preset options were:

1. **Well above**: ~ 66% more than typical
2. **Above**: ~ 33% more than typical
3. **Typical**: ~ 0% from typical
4. **Below**: ~ 33% less than typical
5. **Well below**: ~ 66% less than typical

Steps were estimated using mobile data, wearable devices, four preset levels (2500–10,000 steps), or manual entry option (steps per day)—data were used to calculate non-exercise activity thermogenesis (NEAT) [16].

The five preset options were:

1. **High**: ~ 10,000 steps
2. **Elevated**: ~ 7500 steps
3. **Moderate**: ~ 5000 steps
4. **Low**: ~ 2500 steps

Exercise estimates could be imported from wearable devices, entered manually or calculated using a guided wizard based on Metabolic Equivalent of Task (MET) codes [17]—data were used to calculate exercise activity thermogenesis (EAT). For steps and exercise, participants were encouraged to use device-based inputs to minimise bias. However, multiple input modes were permitted to ensure continuity in free-living conditions (e.g. device failure or missing data). All values were treated as approximate behavioural signals and interpreted within the ENHANCE framework rather than as precise measures of energy intake or energy expenditure.

### Daily energy balance calculation

Daily EB was calculated as follows:

- EB = EI − EE
- EI = Typical EI × Self-reported day modifier. Typical intake was estimated during onboarding from typical EE. This approach is novel, and balances analytical accuracy with real-world practicality.
- EE = REE + TEF + NEAT + EAT. Resting energy expenditure (REE) was calculated using the Mifflin–St Jeor equation [18]. Thermic effect of feeding (TEF) was estimated at 10% of EI [3]. NEAT was estimated by converting daily step counts from mobile, wearable, or self-reported inputs into walking duration and applying standard MET-based energy expenditure equations, with REE subtracted to isolate non-exercise activity thermogenesis (see Supplementary Materials for full calculations). EAT was imported from manufacturer wearable data or estimated using MET codes.

### Weight trend generation and analysis

Five weight change trends were generated to support the analysis, each offering a different perspective on EB:

1. **Raw trend**: Unprocessed body weight data collected each morning from the Wi-Fi scales. Although objective, these measurements vary with natural hydration shifts and data anomalies, making them misleading if interpreted directly as fat weight change.
2. **Smoothed trend**: Generated by removing outliers from raw data using an Interquartile Range (IQR) method (IQR × 1.5) and applying a simple moving average (±3 days). This reduces noise while retaining directional trajectories, providing a cleaner reference for further processing.
3. **Piecewise trend**: Segmented the smoothed trend into three linear phases, each representing a consistent rate of change: pre-Christmas (15–23 December), festive peak (24 December–1 January), and post-New Year (2–10 January). These segments provided a benchmark to evaluate alternative trends.
4. **Predicted trend**: Calculated from self-reported EB values (EI − EE) to estimate daily fat weight change—estimated at 1 kg per 7700 kcal EB [19]. While unaffected by hydration shifts, it is influenced by reporting error and bias. The prediction serves as an input for further processing, rather than an exact estimate of fat change.
5. **Corrected trend**: Generated by aligning predicted and smoothed trends to enhance results. A ± 5-day weighted average from the smoothed trend prioritised nearby low-noise values. The result was used to refine the predicted data to produce a corrected trend grounded in user behaviour and biological plausibility, while aligning with observed weight trends.

### Method evaluation

As a novel method, performance was evaluated through trend comparisons to assess improvements in stability, interpretability, and biological plausibility.

1. **Raw vs piecewise trend**: Evaluates how well raw data aligns with the piecewise trend. Large deviations of >±0.35 kg imply noise from hydration shifts or data anomalies [19, 20].
2. **Corrected vs piecewise trend**: Assesses how closely the corrected trend tracks the piecewise, confirming reduced noise without introducing artefacts.
3. **Corrected vs raw trend**: Confirms reduced noise and recovery of coherent weight signals. Suppressing daily fluctuations >±0.35 kg enhances interpretability while preserving longer-term fat weight trends [19, 20].
4. **Signal-to-noise ratio (SNR)**: Calculated as the ratio of overall variability (SD of weight) to short-term fluctuation (SD of daily changes). Higher post-correction SNR indicates a clearer reflection of fat weight change.
5. **Weight trend autocorrelation**: Calculated as the lag-1 Pearson correlation of daily changes, this reflects directional consistency. Higher autocorrelation post-correction suggests more physiologically coherent progression.
6. **Weight trend plausibility**: Biological plausibility was assessed by comparing implied EB to realistic bounds (−100% to +250% of typical EE). The same thresholds were applied post-correction to ensure results remained within plausible limits.

## Results

### Participants and data completeness

Of the 23 participants who enrolled and completed the protocol, 18 were included in the final analysis (Table 1). Five were excluded due to atypical behaviour from illness (*n* = 4) or bereavement (*n* = 1). At the individual level, adherence to daily body weight measurements was high, with a mean of 94.0% (5.1%). A small proportion of measurements were excluded due to lateness (3.2% after midday) or outliers (3.9% outside IQR threshold), retaining 87.4% of possible scans for analysis. Compliance with EB-related self-reporting of eating, steps and exercise estimates was 100%. Calculated individual-level mean net fat weight change during the monitoring phase was +0.8 kg (0.4 kg); mean net EB was +223 kcal/day (130 kcal/day).

**Table 1:** Participant characteristics and baseline measurements.

Characteristic	Value	Notes
Age (years)	39.3 (6.7)	Mean (SD)
Sex	11 M/7 F	Self-reported
BMI (kg/m²)	24.0 (3.1)	Calculated at onboarding
Height (cm)	172.8 (10.8)	Self-reported
Baseline weight (kg)	72.2 (13.6)	Device-measured (blinded)
Baseline duration (days)	5.7 (1.0)	Time in baseline phase
Typical EI (kcal/day)	1888 (325)	Estimated using typical EE

All values are for the final sample (*n* = 18).

*F* female, *M* male.

### Trend performance

**Fig. 1:**
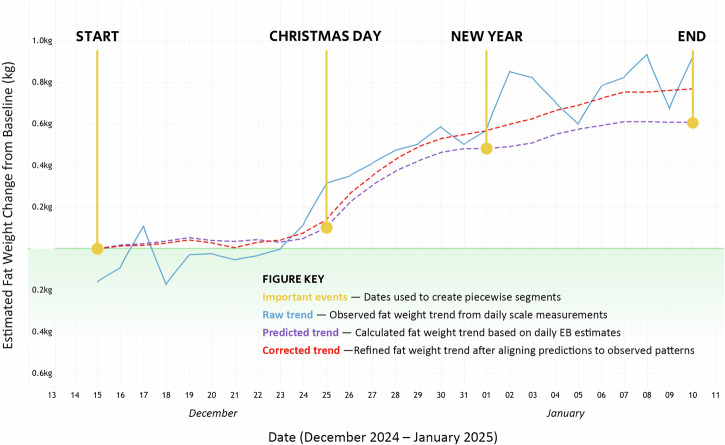
Estimated fat weight change (kg) from baseline across December 2024 to January 2025. The blue solid line shows the raw trend from daily scale measurements, the purple dashed line the predicted trend based on daily EB estimates, and the red dashed line the corrected trend after alignment to observed patterns. Yellow vertical lines and markers indicate important events (start, Christmas Day, New Year, end) used to define piecewise segments.

1. **Raw trend**: Group-level signal quality was low (SNR: 2.69), and autocorrelation was negative (−0.25), indicating limited coherence and poor behavioural interpretability. Daily changes in raw weight were highly volatile, with an individual-level mean of 0.62 kg (0.22 kg), often masking meaningful physiological trends (Fig. 1). Individual-level false direction reversals—where raw weight changes contradicted reported EB, reducing interpretability—occurred on 38.9% (6.7%) of days. Biological plausibility was low, with only 57% of days within realistic bounds (−100% to +250% of typical EE).
2. **Piecewise trend**: Group-level piecewise analysis identified three segments, each with distinct fat weight trends: a modest rise pre-Christmas (+4.3 g/day), a sharp increase during the festive peak (+58.2 g/day), and modest gain post-New Year (+14.9 g/day). The corrected trend aligned more closely with these segments than either raw or predicted.
3. **Predicted trend**: Group-level predicted trajectory explained 90.4% of the variance in smoothed trends (R²: 0.904), showing high internal consistency. Errors were low: mean absolute error (MAE) 93 g (63 g), root mean square error (RMSE) 112 g, and mean bias error (MBE) − 21 g. This accuracy and stability justified its use by the correction method.
4. **Corrected trend**: The correction method refined daily EB estimates by aligning predicted and smoothed trends using a proximity- and noise-weighted smoothing window. This improved stability, interpretability and biological plausibility. Corrected values showed reduced individual-level variability in daily changes: 98 g (48 g); and improved group-level signal quality – SNR: 9.52; and stronger autocorrelation: 0.87—all indicative of a more coherent behavioural signal. Compared to predicted and raw, group-level corrected trends showed the lowest deviation from piecewise (MAE: 102 g, 77 g, and 46 g, respectively), and the highest alignment (r = 0.88)—see Fig. 1 for visual comparisons. At the individual level, false direction reversals were eliminated. The final EB corrections were modest: the mean adjustment was +41 kcal/day (189 kcal/day)—just 2% (6.3%) of reported intake. Daily corrections exceeding 30% were rare, occurring on just 7.8% (8.4%) of days—supporting the method’s restraint and interpretability. Additional comparisons are shown in Table 2.

**Table 2:** Performance summary (individual-level).

*Trend comparisons*	Raw	Predicted	Corrected
R² vs piecewise (Mean ± SD)	–0.6 (1.1)	–1.9 (5.0)	0.6 (0.3)
% of participants MAE < 250 g (vs piecewise)	22%	28%	83%
% of participants MAE < 150 g (vs piecewise)	6%	0%	44%
% of biological plausibility days	57%	100%	100%
% of false direction reversal days (vs predicted)	38.9 (6.7%)	—	0%

*EB corrections*	Mean ± SD	Notes	
EI uncorrected (kcal/day)	2282 (455)	Self-reported	
EI correction (kcal/day)	+41 (198)	Realistic corrections	
EI correction (%)	+1.9% (6.3%)	Minimal corrections	
Days with >30% correction	7.8 (8.4)	Restrained corrections	

### Questionnaire findings

Post-study questionnaires (*n* = 18) indicated high user acceptability and low burden (Table 3). Confidence in self-estimates was high across eating, steps, and exercise. Participants reported negligible behavioural reactivity or impact on Christmas enjoyment.

**Table 3:** Questionnaire findings.

No	Question	Result	Scale
	**Adherence and feasibility**		
1	How much of a burden was it to stand on the device each day?	1.8	1 – Minimal Effort
			5 – Significant Effort
2	How much of a burden was it to complete your daily estimates?	1.8	1 – Minimal Effort
			5 – Significant Effort
	**Usability and confidence**		
3	How confident were you in estimating your eating using the app?	3.2	1 – Not confident
			5 – Very confident
4	How confident were you in estimating your steps using the app?	3.8	1 – Not confident
			5 – Very confident
5	How confident were you in estimating your exercise using the app?	3.8	1 – Not confident
			5 – Very confident
	**Behavioural reactivity**		
6	How much did participating in this study affect your eating choices?	1.6	1 – Not at all
			5 – Significantly
7	Did participating in this study make you eat less than normal?	11%	Yes/No
8	How much did participating in this study affect your exercise choices?	1.4	1 – Not at all
			5 – Significantly
9	Did participating in this study make you exercise more than normal?	0%	Yes/No
10	Did participating in this study impact your enjoyment of Christmas?	6%	Yes/No

## Discussion

### Principal findings

This study evaluates the ENHANCE framework and a novel low-burden method for estimating daily EB using smart devices and minimal self-report. Tested during the demanding festive period, it achieved high adherence and low participant burden. Raw weight data were too volatile and behaviourally implausible for effective use in AI-based coaching. Despite intentionally approximate EB inputs, predicted trends aligned closely with piecewise segments, providing a credible basis for correction. Aligning predicted trends with device measurements produced corrected values that more accurately tracked observed weight changes. This pragmatic design balances scientific rigour with real-world feasibility. By improving stability, interpretability, and biological plausibility, this method offers a superior EB input for AI-driven coaching.

### Strengths of study design

The study design prioritised ecological validity and feasibility without compromising data quality. By avoiding intrusive food tracking and blinding participants to feedback, it ensured naturalistic behaviour and sustained adherence during a disruptive and high-risk period for fat gain [21].

### Limitations and considerations

Firstly, in the absence of laboratory body composition measurements, weight changes were interpreted as shifts in water or fat only, not muscle—reflecting the low likelihood of meaningful muscle gain over short timescales. Second, the sample size was relatively small; larger cohorts will be needed in future studies to assess generalisability of these preliminary findings.

### Implications for health research and personalised healthcare

This method represents a foundational step toward a new paradigm in EB tracking. It demonstrates that personalised health insights can be derived from lightweight, behaviourally passive inputs when enhanced by data processing. Crucially, the approach is both automated and capable of real-time operation, making it well suited for integration within AI coaching platforms. Its ability to track energy dynamics during lifestyle disruptors (e.g. holidays) with high acceptability has important implications for intervention design, public health surveillance, and long-term digital coaching [10]. Future research should prioritise larger cohorts and integration of machine learning to enable adaptive models that learn individual patterns and variability, to provide superior EB tracking and feedback.

## Conclusion

This study introduces the novel ENHANCE framework and evaluates its application for enhanced EB tracking, implemented by combining smart device data with minimal self-reporting. Evaluated during the demanding festive period, it achieved high adherence and low burden while improving accuracy, interpretability, and biological plausibility. By treating self-report as a signal—not ground truth—this method mirrors the challenges faced by health professionals working with imperfect data [22]. Removing the need for burdensome food logs makes this method uniquely suited to free-living use. Automated and scalable through Twists and similar platforms, it can deliver a pioneering capability to support personalised AI-based coaching in real-world settings. Future work should include larger cohorts and explore adaptive models that learn from individual variability—unlocking truly personalised AI coaching.

## Supplementary information

Supplementary Material

## Data Availability

The datasets generated during the current study are not publicly available due to data privacy restrictions. However, anonymised data are available from the corresponding author on reasonable request.
