# Supplementary material for: Novel energy balance tracking to support personalised AI health coaching: a real-world evaluation of the ENHANCE framework
Source: Int J Obes (Lond). 2026 May 12;50(8):1846–51. doi: 10.1038/s41366-026-02100-8 (PMC13461338; doi:10.1038/s41366-026-02100-8)
Supplement: Supplementary file 1 [file 41366_2026_2100_MOESM1_ESM.docx]

**Supplementary Materials**

**Supplementary Methods: Step-Derived NEAT Estimation**

To support energy balance calculations, daily step counts were converted into estimates of non-exercise activity thermogenesis (NEAT). This method was intentionally approximate and used as a behavioural signal within the ENHANCE framework, rather than as a precise measure of energy expenditure.

Daily step count was converted to walking duration using a mean cadence of 76 steps per minute, representing normal-pace walking in free-living adults (1). Gross energy expenditure was then estimated using a MET value of 3.0 for normal-pace walking, as reported in the Compendium of Physical Activities (2). Resting energy expenditure for the same time period was subtracted to isolate NEAT.

**Full calculation**

1. *Walking time (minutes per day)* = Daily steps ÷ 76
2. *Gross energy (kcal per day)* = MET × 3.5 × body weight (kg) ÷ 200 × walking time (minutes per day) (3)
   a. MET − The metabolic equivalent of the activity
   b. 3.5 – Represents resting oxygen consumption in mL/kg/min
   c. 200 – Conversion factor to turn oxygen consumption into kcal/min
3. *NEAT (kcal per day)* = Gross energy (kcal per day) − [REE (kcal per minute) × walking time (minutes per day)]

**References**

1. Dall PM, McCrorie PRW, Granat MH, Stansfield BW. Step accumulation per minute epoch is not the same as cadence for free-living adults. Med Sci Sports Exerc. 2013;45(10):1995–2001.
2. Compendium of Physical Activities [Internet]. Columbia (SC): University of South Carolina; c2011 [cited 2026 Jan 5]. Available from: https://pacompendium.com/
3. American College of Sports Medicine. ACSM’s Guidelines for Exercise Testing and Prescription. 11th ed. Philadelphia: Wolters Kluwer; 2021.
